# Global Metabolite Profiling of Synovial Fluid for the Specific Diagnosis of Rheumatoid Arthritis from Other Inflammatory Arthritis

**DOI:** 10.1371/journal.pone.0097501

**Published:** 2014-06-02

**Authors:** Sooah Kim, Jiwon Hwang, Jinhua Xuan, Young Hoon Jung, Hoon-Suk Cha, Kyoung Heon Kim

**Affiliations:** 1 Department of Biotechnology, Korea University Graduate School, Seoul, Republic of Korea; 2 Samsung Medical Center, Sungkyunkwan University School of Medicine, Seoul, Republic of Korea; Yonsei University, Republic of Korea

## Abstract

Currently, reliable biomarkers that can be used to distinguish rheumatoid arthritis (RA) from other inflammatory diseases are unavailable. To find possible distinctive metabolic patterns and biomarker candidates for RA, we performed global metabolite profiling of synovial fluid samples. Synovial fluid samples from 38 patients with RA, ankylosing spondylitis, Behçet's disease, and gout were analyzed by gas chromatography/time-of-flight mass spectrometry (GC/TOF MS). Orthogonal partial least-squares discriminant and hierarchical clustering analyses were performed for the discrimination of RA and non-RA groups. Variable importance for projection values were determined, and the Wilcoxon-Mann-Whitney test and the breakdown and one-way analysis of variance were conducted to identify potential biomarkers for RA. A total of 105 metabolites were identified from synovial fluid samples. The score plot of orthogonal partial least squares discriminant analysis showed significant discrimination between the RA and non-RA groups. The 20 metabolites, including citrulline, succinate, glutamine, octadecanol, isopalmitic acid, and glycerol, were identified as potential biomarkers for RA. These metabolites were found to be associated with the urea and TCA cycles as well as fatty acid and amino acid metabolism. The metabolomic analysis results demonstrated that global metabolite profiling by GC/TOF MS might be a useful tool for the effective diagnosis and further understanding of RA.

## Introduction

Rheumatoid arthritis (RA) is a chronic autoimmune disease characterized by synovial proliferation and damage of the affected joints. In spite of current treatment advances including the use of tumor necrosis factor-α (TNF-α) inhibitors, early diagnosis of RA using reliable biomarkers is important for early intervention. Rheumatoid factor (RF), a well-known biomarker for RA, is not useful for specific diagnosis of RA because RF is also detected in various other rheumatic (other than RA) and nonrheumatic disorders such as infection and malignancy, and even in normal individuals [1], [2]. Anti-citrullinated protein antibodies (ACPA) have recently received much attention as a valuable tool to differentiate RA from other kinds of arthritis in the 2010 American College of Rheumatology/European League Against Rheumatism (ACR/EULAR) classification criteria [3], [4]. However, not all RA patients are seropositive for ACPA, and the 2010 ACR/EULAR classification criteria does not satisfactorily rule in RA for patients with seronegative arthritis, especially involving only one joint. Therefore, more reliable biomarkers with diagnostic capabilities are still needed for RA.

Recently, omics technologies such as genomics, transcriptomics, proteomics, and metabolomics have been increasingly exploited for the discovery of disease biomarkers, including those for RA. Genomics has clearly revealed differences between ACPA-positive and ACPA-negative diseases [5]. In addition, transcriptomics has been used to discover immunity and defense-related genes in RA patients and to predict the efficacy of the anti-TNF-α biologic agent, infliximab, in RA patients [6], [7]. Metabolomics, which is a non-targeted analysis of global changes of the complete set of metabolites in organisms [8], has shown its potential in the discovery in disease biomarkers [9]–[12]. Because metabolite profile changes can be indicative of a disease state [13]–[15], metabolomics may be a powerful tool for discovering new biomarkers for diseases. Recently, the application of metabolomics to plasma samples was successful in finding metabolic discrimination and potential biomarkers for RA by using nuclear magnetic resonance spectroscopy (NMR) [16], gas chromatography/mass spectrometry (GC/MS), and liquid chromatography/mass spectrometry (LC/MS) [17]. However, to date, reliable biomarkers of RA that discriminate RA from other inflammatory arthritis have not been identified using metabolomics.

Synovial fluid is a body fluid that provides nutrition and lubrication to the articular cartilage. In the pathological joint, the amount of synovial fluid is higher than normal, and a high number of inflammatory cytokines and immune cells are present in the synovial fluid [7]. Thus far, although synovial fluid is the direct medium for the pathological products of RA, no study has examined the changes in metabolism of RA synovial fluid, and biomarkers for RA have not been discovered using synovial fluid. In the present study, in order to find potential biomarkers for RA, discriminating from other kinds of inflammatory arthritis except for septic arthritis (i.e., ankylosing spondylitis (AS), Behçet's disease (BD), and gout), metabolite profiling of synovial fluid from the patients with inflammatory arthritis was performed using gas chromatography/time-of-flight mass spectrometry (GC/TOF MS). These biomarker candidates were verified by multivariate statistical analyses in comparison with other kinds of inflammatory arthritis.

## Materials and Methods

### Human synovial fluid collection and patients

Among patients visiting the rheumatology clinic at the Samsung Medical Center in Seoul, Korea between July 2000 and September 2007, 77 patients who received arthrocentesis were retrospectively screened. Patients with osteoarthritis or a septic condition were excluded from the screening, and thus 38 patients who were diagnosed with RA, ankylosing spondylitis (AS), Behçet's disease (BD), and gout were enrolled in our study. Medical records of the 38 patients were reviewed for age, gender, duration of disease, and laboratory data along with the disease category such as RF, ACPA, fluorescent anti-nuclear antibody, and human leukocyte antigen B27 (HLA-B27). Fulfillment of the above criteria was assessed following the 1987 ACR and 2010 ACR/EULAR classification criteria for RA, the 1984 modified New York criteria, the Assessment of SpondyloArthritis international Society (ASAS) classification criteria for axial spondyloarthritis, and the criteria of the 1990 International Study Group for BD. For gout, the presence of monosodium urate (MSU) crystals was examined in joint fluid. Radiographic findings for the involvement of sacroiliac joints in AS and BD were evaluated, and bony erosion with overhanging edges was checked for gout patients. Following disease categorization, treatment data were obtained for previous uses of non-steroidal anti-inflammatory drugs (NSAIDs) and disease-modifying anti-rheumatic drugs (DMARDs) or uric acid lowering treatment (ULT). In addition, the history of intraarticular steroid injection was investigated.

Synovial fluid samples were obtained from arthrocentesis for the sake of the clinical diagnosis of arthritis. This aspirated synovial fluid was routinely analyzed by examining the white blood cell count, polarizing microscopy, the Gram staining and culture, fungus culture, and acid-fast bacteria staining and culture. The final diagnosis was made by experienced rheumatologists. Synovial fluid samples were collected and stored at −80°C. To identify presumed biomarkers for RA, samples were divided into 2 groups: RA versus non-RA including AS, BD, and gout. The study was carried out in accordance with the Helsinki Declaration and approved by the Institutional Review Board of Samsung Medical Center, Seoul, Korea. All subjects were provided with written informed consent prior to study enrollment.

### Patient characteristics

Synovial fluid samples from 38 patients with inflammatory arthritis were analyzed as RA (13 samples), AS (7 samples), BD (5 samples), and gout (13 samples), and their baseline characteristics are summarized in Table 1. The ages of patients with RA (44.2±10.7) and non-RA (42.1±10.3) did not significantly differ at a significance level of 0.05. Among them, 10 samples were obtained through diagnostic arthrocentesis, whereas other samples were obtained for therapeutic purposes. None of the diagnostic samples were positive for microbial culture. Sacroiliac joints were affected in all AS patients. Five patients with gout had typical erosive lesions as determined from the radiographs, and MSU crystals were confirmed in synovial fluid samples of 7 patients. All RA patients had a history of receiving DMARDs except one patient who was enrolled during the initial presentation of RA. Five of 7 AS patients and 2 of 5 BD patients were prescribed DMARDs before arthrocentesis. Of the 13 patients with gout, 9 had ULT and 10 had received colchicines before enrollment.

**Table 1:** Baseline characteristics of RA and non-RA groups.

	RA (n = 13)	Non-RA (n = 25)		
		AS (n = 7)	BD (n = 5)	Gout (n = 13)
Age, mean ± SD years	44.2±10.7	35.4±10.7	41.6±12.5	45.9±7.9
Female, no. (%)	13 (100)	3 (42.9)	2 (40.0)	0 (0.0)
Disease duration, years	6.5±6.3	3.1±3.3	6.3±7.9	7.9±2.7
RF, no. of positive/tested (%)	13 (100)	0/5 (0.0)	1/3 (33.3)	0/7 (0.0)
ACPA, no. of positive/tested (%)	3/3 (100)	n.a.	n.a.	n.a.
FANA, no. of positive/tested (%)	n.a.	0/3 (0.0)	0/2 (0.0)	0/2 (0.0)
HLA-B27, no. of positive/tested (%)	n.a.	6/6 (100.0)	n.a.	n.a.
Fulfillment of criteria, no. of positive/tested (%)				
1987 ACR	12/13 (92.3)	n.a.	n.a.	n.a.
1984 modified NY	n.a.	7/7 (100.0)	1/5 (20.0)	n.a.
2010 ACR/EULAR	13/13 (100.0)	n.a.	n.a.	n.a.
ASAS axial	n.a.	7/7 (100.0)	n.a.	n.a.
Previous NSAID, no. of positive/tested (%)	12/13 (92.3)	22/25 (88.0)	2/5 (40.0)	13/13 (100.0)
Previous intraarticular steroid injection, no. or no. of positive/tested (%)	10/13 (76.9)	4/7 (57.1)	3/5 (60.0)	3/13 (23.1)

ACPA, anti-CCP antibody; ACR, The American College of Rheumatology classification criteria of RA; ACR/EULAR, The American College of Rheumatology/European League Against Rheumatism classification criteria for RA; AS, ankylosing spondylitis; ASAS axial, Assessment of SpondyloArthritis international Society classification criteria for axial spondyloarthritis; BD, Behçet's disease; FANA, fluorescent anti-nuclear antibody; HLA-B27, human leukocyte antigen B27; modified NY, Modified New York criteria for the diagnosis of AS; n.a, not applicable; non-RA, non-rheumatoid arthritis including ankylosing spondylitis, Behçet's disease, and gout; Previous NSAID, previously use of non-steroidal anti-inflammatory drug; RA, rheumatoid arthritis; RF, rheumatoid factor.

### Metabolite sample preparation

Metabolite extraction from synovial fluid was conducted using 80% (v/v) methanol at −20°C according to a previously described procedure with a slight modification [18]. Synovial fluid samples were thawed on ice for 3 min and then centrifuged at 500×*g* at 4°C for 5 min to remove cells and debris. The supernatant from the centrifuged synovial fluid was mixed with 80% (v/v) methanol at −20°C for metabolite extraction, and this mixture was vortexed for 3 min and then centrifuged at 16100×*g* for 5 min at 4°C. The supernatant was then completely dried in a vacuum concentrator (Labconco, Kansas City, MO). To eliminate lipids and waxes, the metabolite extract was re-extracted with 500 µL of an aqueous acetonitrile solution (acetonitrile:water  = 1:1, v/v) at 0°C. After centrifugation at 16100×*g* for 5 min, the supernatant was collected and concentrated to dryness. The dried metabolite was derivatized with 5 µL of methoxyamine hydrochloride in pyridine (40 mg/mL; Pierce, Rockford, IL) for 90 min at 30°C and 45 µL of *N*-methyl-*N*-(trimethylsilyl) trifluoroacetamide (Fluka, Buchs, Switzerland) was added for 30 min and 37°C. Subsequently, a mixture of fatty acid methyl esters as retention index markers was added to the derivatized sample.

### Metabolite analysis

An Agilent 7890A GC (Hewlett-Packard, Atlanta, GA) coupled to a Pegasus HT TOF MS (Leco, St. Joseph, MI) was used for the analysis of derivatized metabolite samples. The derivatized extract (1 µL) was injected into the GC in splitless mode. An RTX-5Sil MS capillary column (30 m length, 25 mm inner diameter, and 0.25 mm film thickness; Restek, Bellefonte, PA) and an additional 10-m long integrated guard column were used for GC separation. The sample was initially held at a constant temperature of 50°C for 1 min, after which it was ramped to 330°C at 20°C/min and then finally held for 5 min. The transfer line temperature was set at 280°C. Mass spectra were acquired in a scanning range of 85–500 *m/z* at an acquisition rate of 10 spectra/sec. The ionization mode was subjected to electron impact at 70 eV with an ion source temperature set at 250°C. GC/TOF MS data were preprocessed by Leco ChromaTOF software (version 3.34; Leco) by using automated peak detection and mass spectral deconvolution. Preprocessed MS data were processed using BinBase, an in-house programmed database for the identification of metabolites, as described previously [19], [20]. The abundance of each identified metabolite was obtained by normalizing the peak intensity of each metabolite using the median of sums of peak intensities of all the identified metabolites in each sample [21], [22].

### Statistical analyses and validation

As the statistical analyses of metabolite profiles of synovial fluid from the RA and non-RA (AS, BD, and gout) groups, univariate analysis [19], [20], [23], orthogonal partial least squares discriminant analysis (OPLS-DA), hierarchical clustering analysis (HCA) [24], and receiver operating characteristic (ROC) curve analysis were performed. To obtain maximal covariance between the measured data and the response variable, OPLS-DA was performed using SIMCA-P+ (version 12.0; Umetric AB, Umea, Sweden). Seven-fold internal cross validation and external validation were also conducted using SIMCA-P+. For the external validation, RA patients and non-RA patients were randomly collected from another cohort. The mean age of 6 RA patients (five female and one male) was 66.5 years, and that of 11 non-RA patients (one female and ten male) consisting of 4 AS patients, 4 BD patients and 3 gout patients was 32.5 years. Hierarchical clustering analysis (HCA) was performed using MultiExperiment Viewer for visualization and organization of metabolite profiles [24]. Statistica (version 7.1; StatSoft, Tulsa, OK) was used for univariate analysis [19], [20], [23]. A further diagnostic property was deduced by receiver operating characteristic (ROC) curve analysis using MedCalc software (Broekstraat, Mariakerke, Belgium).

## Results

### Metabolite profiles of RA and non-RA groups

A total of 38 synovial fluid samples of inflammatory arthritis including RA, AS, BD, and gout were analyzed by GC/TOF MS. After deconvolution, 105 metabolites were identified across the synovial fluid samples of 38 patients, which were classified into the following chemical classes: sugars and sugar alcohols (25%), amino acids (21%), fatty acids (16%), organic acids (16%), amines (9%), phosphates (8%), and miscellaneous (Table S1).

**Figure 1:**
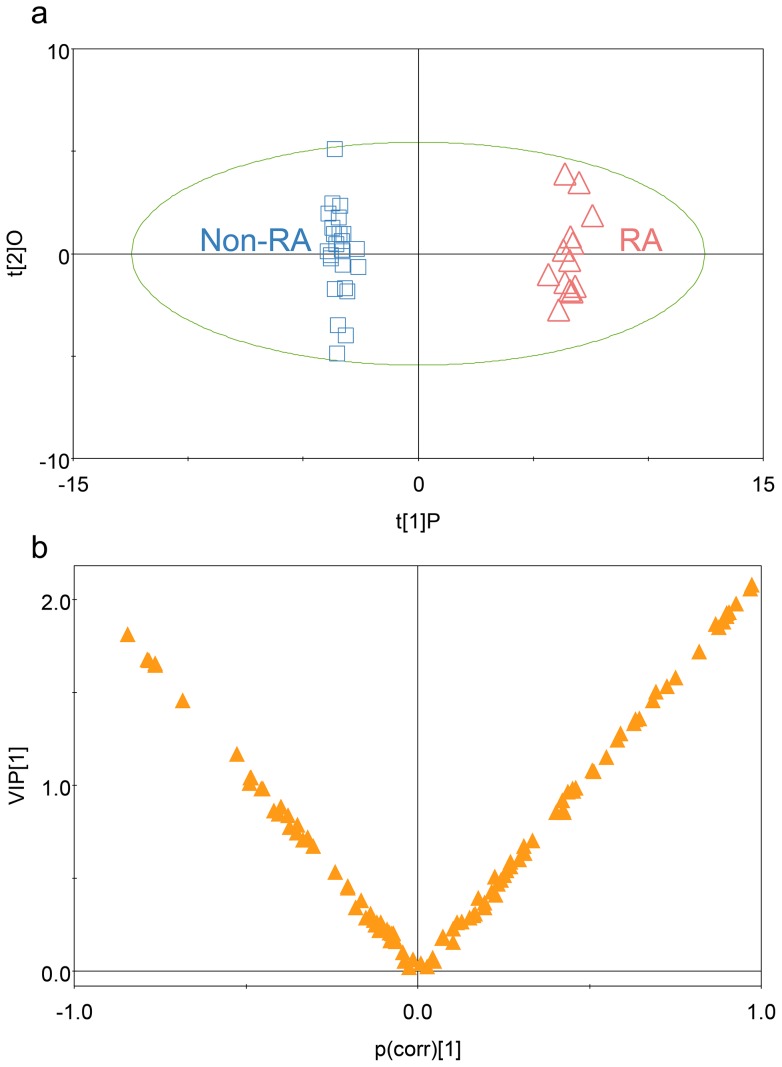
OPLS-DA of the metabolite profiles of RA and non-RA groups. (a) Score plot of the OPLS-DA model for RA and non-RA groups (t[1]P, score of the non-orthogonal component; t[2]O, score of the orthogonal component). (b) V-plot with p(corr) and VIP values of 105 metabolites. The metabolites with p(corr) <0 were those decreased in RA groups while the metabolites with p(corr) >0 were those increased in RA groups.

Since principal component analysis (PCA) showed only slight discrimination between RA and non-RA groups (R^2^X = 0.34, Q^2^ = 0.20) in a preliminary study (data not shown), OPLS-DA was employed in this study. OPLS-DA successfully minimized the possible contribution of intergroup variability and further increased the discrimination between the RA and non-RA groups compared to the results obtained by the PCA. As shown in Figure 1a, metabolite profiles of the RA and non-RA groups were distinctively separated on the score plot of OPLS-DA. The OPLS-DA model established with one predictive component and two orthogonal components generated the explained variation values: 0.36 of R^2^X and 0.99 of R^2^Y and the predictive capability: Q^2^ of 0.97. These high value parameters indicated the excellence in modeling and prediction with good discrimination between the RA and non-RA groups since OPLS-DA models with the parameters higher than 0.5 are considered to be satisfactory in explanatory and predictive capabilities [25]. To validate the OPLS-DA model, the PLS-DA model with the same number of components was used. All permuted R^2^ values to the left were lower than the original point to the right, and the Q^2^ regression line had a negative intercept (Figure S1-a). These results strongly indicated that the OPLS-DA models were statistically validated without overfitting of the original model since the intercept of Q^2^ was less than 0.05. In addition, 6 RA patients and 11 non-RA patients collected from another cohort were predicted to be in correct classes (Figure S1-b).

**Figure 2:**
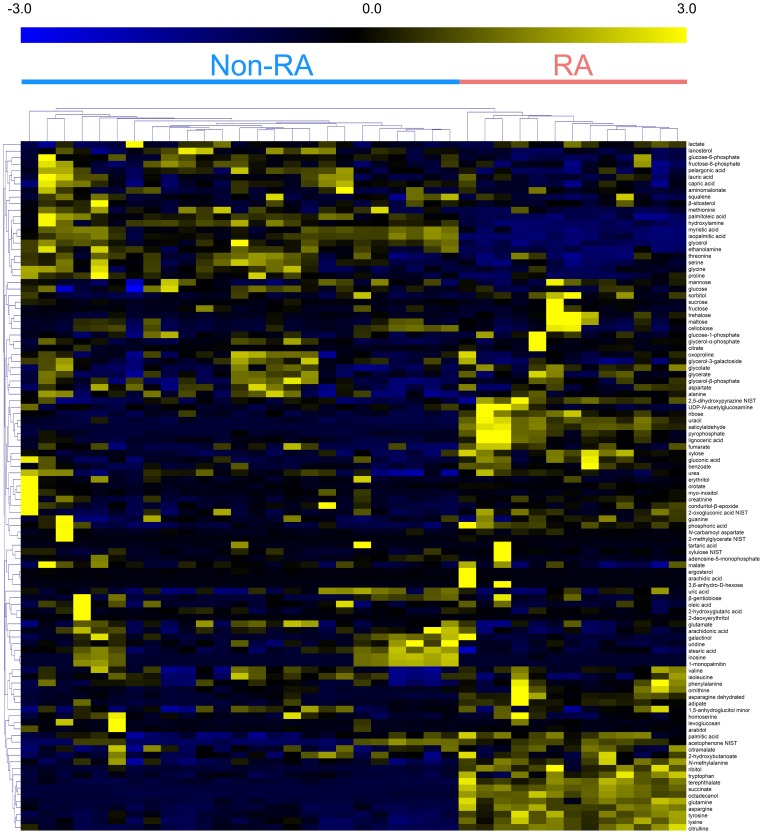
HCA of 105 metabolites from synovial fluid samples of RA and non-RA patients. Each column and row represents a disease and an individual metabolite, respectively.

A total of 105 identified metabolites were clustered and visualized by the HCA using the Euclidean distance and the average linkage method to determine possible variations in the metabolite profiling of the RA and non-RA groups. The normalized peak intensity of each metabolite was transformed by unit variance scaling and loaded into a clustered heat map (Figure 2). The higher the abundance of the metabolites, the more yellow in the heat map, and the lower the abundance of the metabolites, the more blue in the heat map. Clustering of the metabolites led to good separation between the RA and non-RA groups. The discrimination of metabolite profiles between the two groups was mainly caused by certain metabolites as shown in Figure 2.

### Identification of biomarkers for RA

Identification of potential biomarker candidates that account for the differentiation of diseases is a necessary step not only for diagnosis but also for better understanding of the functional metabolism in clinical diseases. To screen putative biomarkers for RA, the variable importance for projection (VIP) values from the OPLS-DA model were obtained. Then, the nonparametric Wilcoxon-Mann-Whitney test and the breakdown and one-way analysis of variance (ANOVA) with post hoc Tukey's honestly significant difference (HSD) test led to further testing of the selected metabolites with high VIP values as biomarker candidates for RA.

VIP values were used to rank the contribution of metabolites to the discrimination between the RA and non-RA groups, which are based on weighted coefficients of the OPLS-DA model [25]. Using the p(corr) and VIP values of the 105 metabolites in synovial fluid, a V-plot was constructed (Figure 1b). VIP values and correlation coefficients (i.e., p(corr)) of each metabolites were shown in the V-plot. Metabolites in both terminals of V represented a high contribution to the discrimination of the RA and non-RA groups. In a VIP analysis, VIP values above 1 are considered important since the influence of variables with a VIP >1.0 on the explanation of the Y matrix is above average [25]. In this study, 33 metabolites were found to have VIP values higher than 1, of which 23 metabolites were higher in the RA group, whereas 10 metabolites were higher in the non-RA group.

Next, the Wilcoxon-Mann-Whitney test was employed to evaluate significant differences (*p*<0.01) of metabolite candidates and to eliminate variables without significant differences between the two groups. Because the abundance of ornithine between the RA and non-RA groups was not significantly different at the 99% significance level, ornithine was ruled out from the 33 biomarker candidates. Among the 32 metabolites that passed the Wilcoxon-Mann-Whitney test, the abundances of 22 metabolites, including succinate, octadecanol, asparagine, and terephthalate, were higher in the RA group than in the non-RA group. Meanwhile, the abundances of 10 metabolites, including isopalmitic acid, glycerol, myristic acid, and palmitoleic acid, were lower in the RA group than in the non-RA group.

One-way ANOVA was conducted to select putative biomarkers for the RA group only in comparison with the non-RA group representing other inflammatory arthritis including AS, BD, and gout. A post-hoc Tukey's HSD test at the 99% significance level was then performed to compare the mean values between groups. The following metabolites did not significantly differ in abundance between the RA group and each disease group of AS, BD, and gout in ANOVA and HSD tests: adipate, asparagine dehydrated, 2,5-dihydroxypyrazine NIST, lanosterol, lignoceric acid, *N*-methylalanine, palmitic acid, phosphoric acid, proline, pyrophosphate, serine, and stearic acid. All of these metabolites were eliminated from the putative biomarkers for RA.

The fold changes of the 20 metabolites selected as potential biomarkers to discriminate RA from non-RA are shown in Figure S2. The abundances of succinate, octadecanol, asparagine, terephthalate, salicylaldehyde, glutamine, citrulline, tyrosine, uracil, lysine, ribitol, tryptophan, xylose, and ribose were higher in the RA group than those in the non-RA group. However, the abundances of isopalmitic acid, glycerol, myristic acid, palmitoleic acid, hydroxylamine, and ethanolamine were lower in the RA group than those in the non-RA group. Notably, the fold change of succinate was highest in the RA group, and the fold changes of salicylaldehyde and glutamine were much higher than those of other metabolites in the RA group. The fold changes of the metabolite abundances increased in the RA group ranged from 1.7 to 73.6.

### ROC analysis

**Figure 3:**
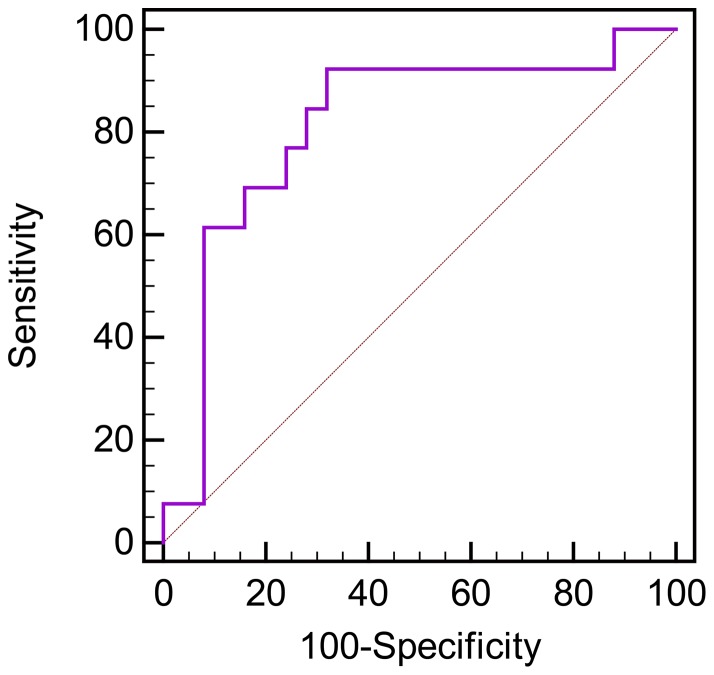
ROC analysis of the predictive power of the 20 combined biomarkers for distinguishing RA and non-RA groups. A sensitivity and specificity were 92.3% and 68.0%, respectively, and the value of AUC was 0.812.

Twenty putative biomarkers of the RA group were selected after employing multiple statistical analyses as described earlier. Prior to clinical utility of the 20 putative biomarkers, validation of the biomarkers is needed. For disease diagnosis, the ROC curve and the area under the ROC curve (AUC) provide a numerical value of the relationship between the specificity and sensitivity of a biomarker. These sensitivity and specificity indicate the probably tests for correctly identifying patients with the disease and without the disease, respectively [26]. An AUC value of 0.5 or less for a biomarker indicates no information and discrimination within the test, thus implying no diagnostic utility of the biomarker, whereas an AUC value of 1.0 indicates perfect prediction of the diagnostic test [27]-[29]. Figure 3 shows the ROC curve analysis for the predictive power of the 20 combined biomarkers of the RA group to discriminate RA from non-RA. A sensitivity of 92.3% and a specificity of 68.0% were obtained from the ROC curve, and the value of AUC was 0.812. Since the 20 putative biomarkers showed the AUC value of greater than 0.8, they were selected as biomarkers of RA (Table 2).

**Table 2:** VIP and AUC values of the metabolites that significantly contribute to the discrimination between the RA and non-RA groups.

Metabolite	VIP value (rank)	*p*-value^a^	AUC^b^
Metabolites with higher abundances in the RA group than in the non-RA group			
succinate	2.09 (1)	<0.0001	1.000
octadecanol	2.07 (2)	<0.0001	1.000
asparagine	1.98 (3)	<0.0001	1.000
terephthalate	1.94 (4)	<0.0001	1.000
salicylaldehyde	1.93 (5)	<0.0001	1.000
glutamine	1.92 (6)	<0.0001	0.997
citrulline	1.91 (7)	<0.0001	1.000
tyrosine	1.89 (8)	<0.0001	1.000
uracil	1.87 (9)	<0.0001	0.997
lysine	1.86 (10)	<0.0001	0.994
ribitol	1.72 (12)	<0.0001	0.985
tryptophan	1.59 (17)	<0.0001	0.883
xylose	1.54 (18)	<0.0001	0.92
ribose	1.51 (19)	<0.0001	0.969

Metabolites with lower abundances in the RA group than in the non-RA group			
isopalmitic acid	1.82 (11)	<0.0001	0.994
glycerol	1.68 (13)	<0.0001	1.000
myristic acid	1.68 (14)	<0.0001	0.985
palmitoleic acid	1.66 (15)	<0.0001	1.000
hydroxylamine	1.65 (16)	<0.0001	1.000
ethanolamine	1.46 (20)	<0.0001	0.963

a *p*-values were determined using the Wilcoxon-Mann-Whitney test.

b Area under the receiver operator characteristics curve.

## Discussion

Recently, the importance of metabolomics for the study of disease biomarkers and metabolism is rapidly increasing [30]–[32]. Zahi et al. reported the branched-chain amino acids to histidine ratio as a novel serum biomarker of osteoarthritis using a metabolomics approach [33]. However, only a few studies have performed non-targeted metabolite profiling of RA on a global scale by using plasma or synovial fluid [16], [17], [34]. Especially, reliable biomarkers of RA distinguished from other inflammatory arthritis such as AS, BD, and gout have not been identified using metabolite profiling in synovial fluids, which is the direct medium showing the state of disease. For example, in a previous study of metabolite profiling of synovial fluid from RA, AS, and gout patients using ^1^H-NMR identifying 35 metabolites, no differences in metabolite profiles were shown between those diseases [34]. In this study, GC/TOF MS was used to find possible biomarkers among metabolites in the synovial fluid of patients with inflammatory arthritis in order to differentiate RA from other – inflammatory arthritis such as AS, BD, and gout by using metabolomics. The metabolite profiles of synovial fluid obtained from RA patients were distinguishable from those of other inflammatory arthritis, in which 20 metabolites were selected and validated as potential biomarkers with the capability of discriminating RA from the non-RA diseases like AS, BD, and gout with 92.3% sensitivity and 68.0% specificity. This is the first report of the discovery of potential biomarkers for RA, which discriminate RA from other inflammatory arthritis, by GC/TOF MS-based metabolomic analysis of synovial fluid.

**Figure 4:**
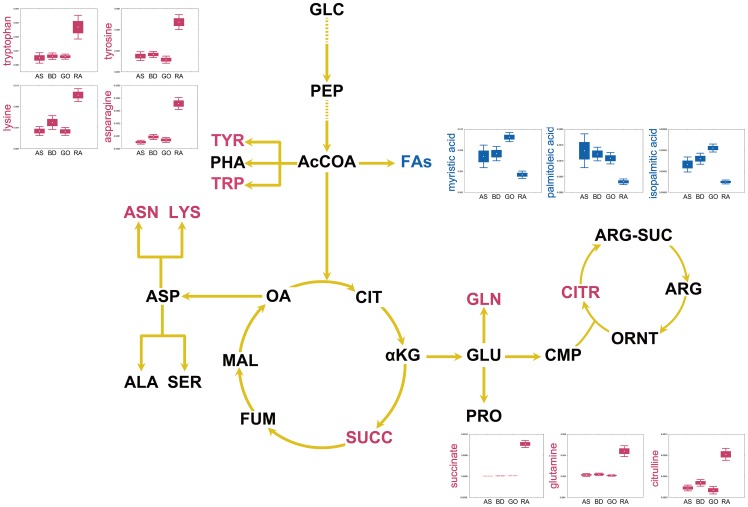
Schematic comparison of the primary metabolisms of RA vs. non-RA groups (AS, BD, and GO). The box and whisker plots indicate the intracellular metabolite levels for each disease group (red, increased in RA; green, increased in non-RA). *AcCOA*, acetyl-CoA; *ALA*, alanine; *ARG*, arginine; *ARG-SUC*, arginine-succinate; *ASN*, asparagine; *ASP*, aspartate; *CIT*, citrate; *CITR*, citrulline; *CMP*, carbamoyl phosphate; *FAs*, fatty acids; *FUM*, fumarate; *GLC*, glucose; *GLN*, glutamine; *GLU*, glutamate; *αKG*, α-ketoglutarate; *LYS*, lysine; *MAL*, malate; *OA*, oxalate; *ORNT*, ornithine; *PEP*, phosphoenolpyruvate; *PHA*, phenylalanine; *PRO*, proline; *SER*, serine; *SUCC*, succinate; *TRP*, tryptophan; *TYR*, tyrosine.

In the present study, 105 metabolites classified into various chemical classes such as amines, amino acids, fatty acids, organic acids, phosphates, and sugars and sugar alcohols were identified by an in-house library. These metabolites are major intermediates of various metabolic pathways, including glycolysis, the TCA cycle, as well as pathways involving amino acid and fatty acid metabolism. The number of metabolites identified from synovial fluid of RA in this study was much higher than that in previous studies [34]. In this study, the metabolite profiles of synovial fluid from RA and non-RA groups were considerably discriminated by OPLS-DA. Following various statistical analyses, 20 metabolites of synovial fluid, including succinate, octadecanol, asparagine, terephthalate, salicylaldehyde, glutamine, citrulline, tyrosine, uracil, lysine, ribitol, tryptophan, xylose, ribose, isopalmitic acid, glycerol, myristic acid, palmitoleic acid, hydroxylamine, and ethanolamine were selected and validated as putative biomarkers for RA, which discriminated from non-RA diseases such as AS, BD, and gout. These metabolites are the major intermediates of the TCA cycle, urea cycle, and fatty acid and amino acid metabolism (Figure 4).

In particular, citrulline synthesized from ornithine and carbamoyl phosphate is a key intermediate of the urea cycle. It is also generated by posttranslational modification of arginine residues by peptidylarginine deiminase [35]. Because citrulline is a major antigenic determinant recognized by RA, ACPAs have been used for the diagnosis of RA and have been established as a useful tool to discriminate RA from other arthritic diseases [36]. Moreover, in this study, the abundances of citrulline and ornithine were significantly higher in the RA group than those in the non-RA group. In the TCA cycle, α-ketoglutarate is a precursor to such amino acids as glutamate, glutamine, proline, and arginine. Oxaloacetate, which is converted from succinate, fumarate, and malate, is also a precursor to such amino acids as asparagine, methionine, threonine, isoleucine, and lysine [37]. The abundances of asparagine, glutamine, tyrosine, lysine, and tryptophan were higher in the RA group than those in non-RA group. Although α-ketoglutarate and oxaloacetate from the TCA cycle were not identified as metabolites in the present study, the abundances of succinate and fumarate in the TCA cycle were higher in the RA group, as were their derivative amino acids asparagine, lysine, and glutamine. These results indicate that the urea and TCA cycles as well as amino acid metabolism were highly activated in the RA group compared with the non-RA group consisting of AS, BD, and gout patients. In addition to citrulline, succinate, asparagine, glutamine, and lysine can be considered as major biomarkers for RA diagnosis.

Fatty acids are synthesized from acetyl-CoA and play important roles in cellular metabolism. RA is known to be affected by n-3 and n-6 fatty acids. For example, n-3 fatty acids suppress inflammation by reducing TNF-α and interleukin-1β levels in RA patients by competitively inhibiting the production of leukotriene B4 from arachidonic acid [38]. In our study, arachidonic acid (an n-6 fatty acid) was identified, but the level of arachidonic acid between the RA and non-RA groups did not significantly differ at the 99% significance level. Other than arachidonic acid, major fatty acids such as isopalmitic acid, myristic acid, and palmitoleic acid were identified as the significant metabolites in the RA group because their levels were markedly lower in the RA group. These results indicate that the fatty acid metabolism was more activated in the non-RA group than in the RA group.

This study has some limitations in the sample size and gender ratio. Although the sample size was relatively small here, the OPLS-DA model was well validated by the permutation test (Figure S1-a), and the potential biomarkers of RA were also verified by external validation (Figure S1-b) and AUC (Table 2). The gender ratio was not controlled in each group in this study, but among the 20 biomarkers of RA found from 13 RA and 25 non-RA patients without gender ratio control, 14 metabolites reappeared as the biomarkers of RA from 13 RA and 5 non-RA patients with gender ratio control (Table S2). These results agreed with previous reports that the metabolic profiles of RA did not significantly affected by gender [16], [39].

In conclusion, this is the first report on the identification of potential biomarkers for RA using human synovial fluid of RA and non-RA patients by metabolomics for the diagnosis of RA distinguished from other inflammatory arthritis such as AS, BD, and gout. We also demonstrated that metabolic profiling may be a useful tool to discover biomarkers, and envision a holistic view of metabolism for diseases.

## Supporting Information

Figure S1 **OPLS model of the metabolite profiles of RA and non-RA groups. (a) Validation of the OPLS-DA model using 100 permutation test.** Y-axis intercept of R2 and Q2 were 0.514 and −0.231, respectively. (b) Y-predicted scatter plot of the OPLS-DA model validated with RA and non-RA patients from another cohort. Red, RA patients; Blue, non-RA patients; Orange, RA and non-RA patients from another cohort.
(TIF)

Figure S2 **Fold changes of abundances of 20 metabolites in synovial fluid selected as potential biomarkers for RA.** Positive values indicate the increased fold changes in the RA group and negative values the increased fold changes in the non-RA group.
(TIF)

Table S1 Metabolites identified from GC/TOF MS and BinBase analyses of synovial fluid.
(DOC)

Table S2 The potential biomarkers of RA found from metabolite analysis of synovial fluid with and without controlling gender ratios of RA and non-RA patients.
(DOC)
