# Supplementary figures and images for: Global Metabolite Profiling of Synovial Fluid for the Specific Diagnosis of Rheumatoid Arthritis from Other Inflammatory Arthritis
Source: PLoS One. 2014 Jun 2;9(6):e97501. doi: 10.1371/journal.pone.0097501 (PMC4041724; doi:10.1371/journal.pone.0097501)

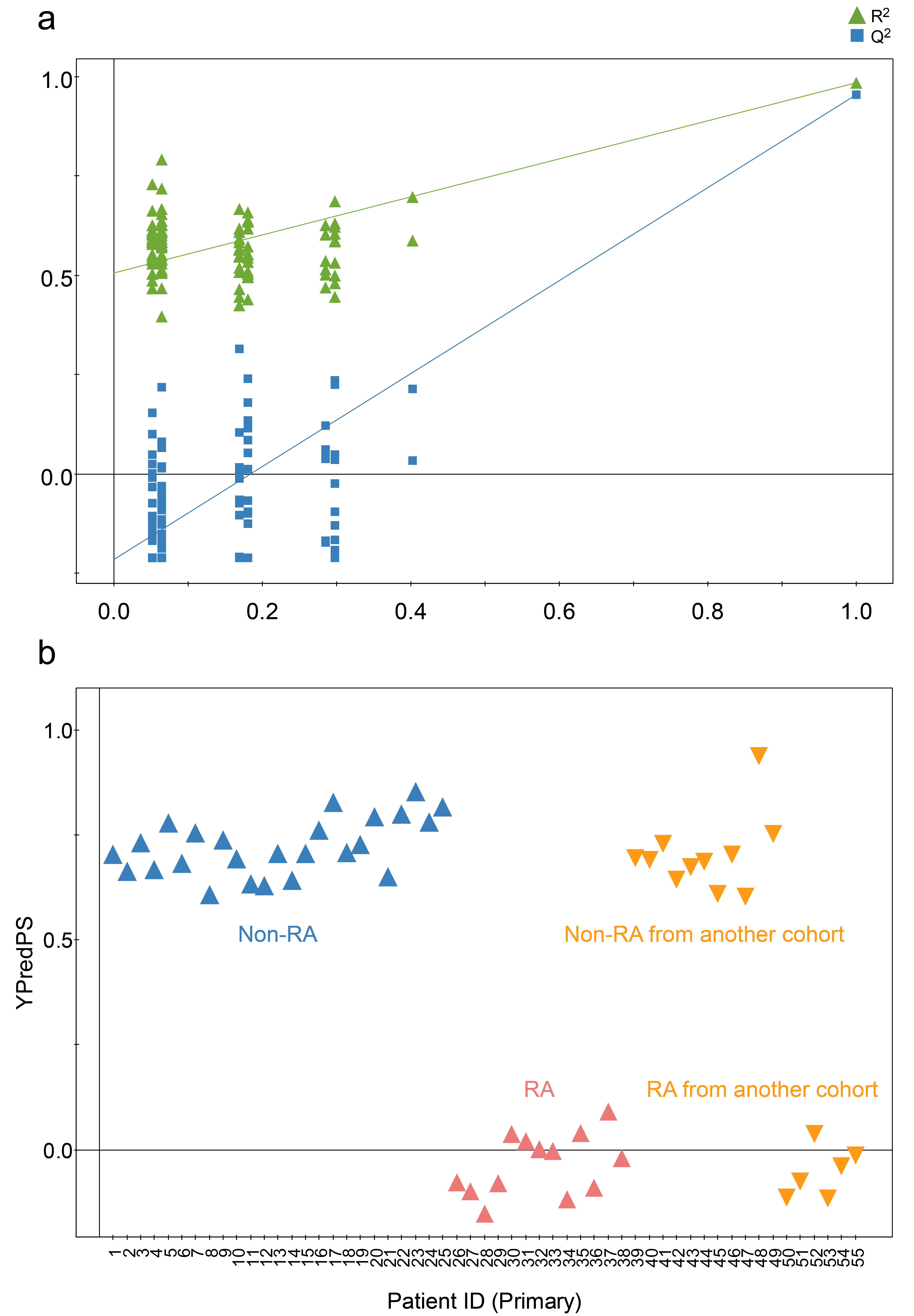

Supplement: Figure S1 — OPLS model of the metabolite profiles of RA and non-RA groups. (a) Validation of the OPLS-DA model using 100 permutation test. Y-axis intercept of R2 and Q2 were 0.514 and −0.231, respectively. (b) Y-predicted scatter plot of the OPLS-DA model validated with RA and non-RA patients from another cohort. Red, RA patients; Blue, non-RA patients; Orange, RA and non-RA patients from another cohort. (TIF) [file pone.0097501.s001.tif]

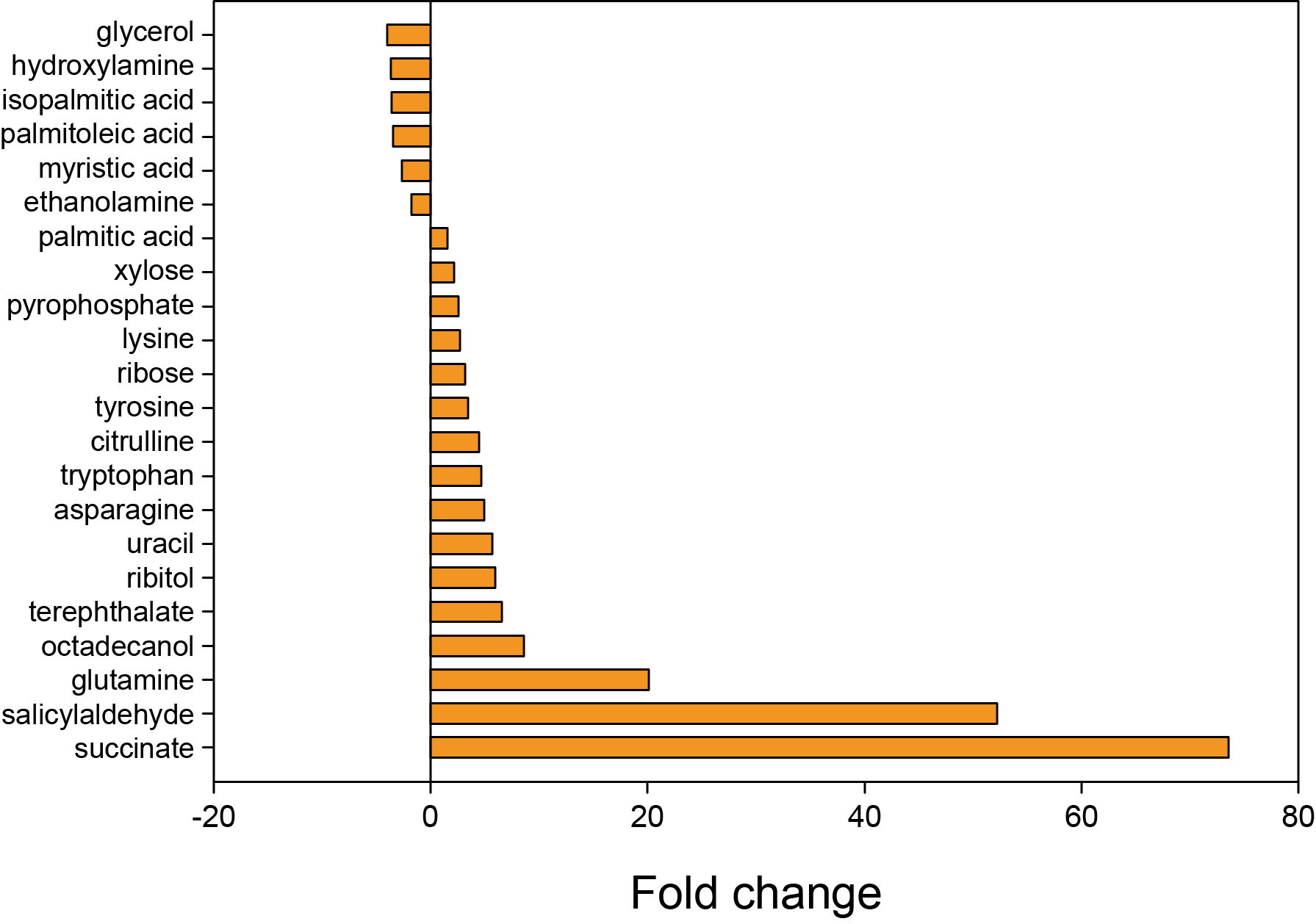

Supplement: Figure S2 — Fold changes of abundances of 20 metabolites in synovial fluid selected as potential biomarkers for RA. Positive values indicate the increased fold changes in the RA group and negative values the increased fold changes in the non-RA group. (TIF) [file pone.0097501.s002.tif]
