# Supplementary material for: Global Metabolite Profiling of Synovial Fluid for the Specific Diagnosis of Rheumatoid Arthritis from Other Inflammatory Arthritis
Source: PLoS One. 2014 Jun 2;9(6):e97501. doi: 10.1371/journal.pone.0097501 (PMC4041724; doi:10.1371/journal.pone.0097501)
Supplement: Table S1 — Metabolites identified from GC/TOF MS and BinBase analyses of synovial fluid. (DOC) [file pone.0097501.s003.doc]

**Table S1.** Metabolites identified from GC/TOF MS and BinBase analyses of synovial fluid.

| Class | Identified metabolite | Retention index | Quant mass |
| --- | --- | --- | --- |
| sugar and | 3,6-anhydro-D-hexose | 602697 | 158 |
| sugar alcohol | 1,5-anhydroglucitol minor | 631996 | 218 |
|  | arabitol | 571650 | 217 |
|  | cellobiose | 942611 | 204 |
|  | 2-deoxyerythritol | 352858 | 117 |
|  | erythritol | 470445 | 217 |
|  | fructose_T^a^ (fructose 1 + fructose 2) | 637600, 642090 | 307 |
|  | galactinol | 1001352 | 204 |
|  | β-gentiobiose | 963984 | 361 |
|  | gluconic acid | 693197 | 333 |
|  | glucose_T^a^ (glucose 1 + glucose 2) | 648900, 659689 | 160 |
|  | glycerol | 342192 | 218 |
|  | glycerol-3-galactoside | 799761 | 204 |
|  | 2-hydroxyglutaric acid | 505173 | 247 |
|  | levoglucosan | 568781 | 204 |
|  | maltose | 944857 | 204 |
|  | mannose | 643834 | 160 |
|  | myo-inositol | 729376 | 305 |
|  | 2-oxogluconic acid NIST^b^ | 541046 | 204 |
|  | ribitol | 574272 | 217 |
|  | ribose | 551695 | 217 |
|  | sorbitol | 666244 | 307 |
|  | sucrose | 913184 | 361 |
|  | trehalose | 946757 | 361 |
|  | xylose | 543829 | 307 |
|  | xylulose NIST^b^ | 551121 | 173 |
| amino acid | alanine_T^a^ (alanine 1 + alanine 2) | 243588, 239999 | 116 |
|  | asparagine | 552180 | 116 |
|  | asparagine dehydrated | 476166 | 243 |
|  | aspartate_T^a^ (aspartate + aspartate minor) | 479698, 432163 | 232 |
|  | *N*-carbamoyl aspartate | 609992 | 257 |
|  | glutamine | 598729 | 156 |
|  | glutamate | 528291 | 246 |
|  | glycine | 368221 | 174 |
|  | homoserine | 443050 | 218 |
|  | isoleucine_T^a^ (isoleucine + isoleucine minor) | 358174, 293914 | 158 |
|  | lysine | 663163 | 156 |
|  | methionine | 483359 | 176 |
|  | *N*-methylalanine | 285385 | 130 |
|  | ornithine | 526294 | 142 |
|  | oxoproline | 486607 | 156 |
|  | phenylalanine | 537197 | 218 |
|  | proline | 364232 | 142 |
|  | serine_T^a^ (serine + serine minor) | 393234 | 204 |
|  | threonine | 408085 | 218 |
|  | tryptophan | 780149 | 202 |
|  | tyrosine | 670302 | 218 |
|  | valine | 312150 | 144 |
| fatty acid | arachidic acid | 855617 | 117 |
|  | arachidonic acid | 833906 | 91 |
|  | capric acid | 451016 | 229 |
|  | ergosterol | 1100979 | 363 |
|  | isopalmitic acid | 698954 | 313 |
|  | lanosterol | 1130233 | 393 |
|  | lauric acid | 546748 | 257 |
|  | lignoceric acid | 975957 | 145 |
|  | 1-monopalmitin | 899896 | 371 |
|  | myristic acid | 632916 | 117 |
|  | octadecanol | 754350 | 327 |
|  | oleic acid | 778361 | 145 |
|  | palmitic acid | 713086 | 313 |
|  | palmitoleic acid | 703584 | 117 |
|  | pelargonic acid | 398295 | 215 |
|  | squalene | 975432 | 121 |
|  | stearic acid | 787169 | 117 |
| organic acid | adipate | 473852 | 111 |
|  | aminomalonate | 454814 | 218 |
|  | benzoate | 339532 | 179 |
|  | citramalate | 455879 | 247 |
|  | citrate | 615691 | 273 |
|  | fumarate | 389305 | 245 |
|  | glycerate | 375499 | 189 |
|  | glycolate | 228746 | 177 |
|  | 2-hydroxybutanoate | 260098 | 131 |
|  | lactate | 216761 | 117 |
|  | malate | 461689 | 233 |
|  | 2-methylglycerate NIST^b^ | 371347 | 219 |
|  | orotate | 584714 | 254 |
|  | succinate | 368843 | 129 |
|  | tartaric acid | 533422 | 292 |
|  | terephthalate | 610506 | 295 |
|  | uric acid | 729944 | 441 |
| amine | citrulline | 620728 | 157 |
|  | creatinine | 502503 | 143 |
|  | 2,5-dihydroxypyrazine NIST^b^ | 396569 | 241 |
|  | ethanolamine | 342547 | 174 |
|  | guanine | 742665 | 352 |
|  | hydroxylamine | 252632 | 146 |
|  | inosine | 896356 | 230 |
|  | UDP-*N*-acetylglucosamine | 622563 | 226 |
|  | uracil | 384920 | 241 |
|  | uridine | 856224 | 245 |
| phosphate | adenosine-5-monophosphate | 1038180 | 315 |
|  | fructose-6-phosphate | 803573 | 315 |
|  | glucose-1-phosphate | 593842 | 217 |
|  | glucose-6-phosphate_T^a^  (glucose-6-phosphate 1 + glucose-6-phosphate 2) | 805229, 808818 | 387 |
|  | glycerol-α-phosphate | 589738 | 299 |
|  | glycerol-β-phosphate | 573347 | 243 |
|  | phosphoric acid | 343727 | 299 |
|  | pyrophosphate | 324646 | 110 |
| miscellaneous | acetophenone NIST^b^ | 243111 | 105 |
|  | conduritol-β-epoxide | 703210 | 318 |
|  | salicylaldehyde | 407083 | 193 |
|  | β-sitosterol | 1128612 | 129 |
|  | urea | 329079 | 171 |

^a^Summed to one value for the case of metabolites with more than one peak.

^b^Metabolites annotated by NIST mass spectral search.
