# Supplementary material for: Global Metabolite Profiling of Synovial Fluid for the Specific Diagnosis of Rheumatoid Arthritis from Other Inflammatory Arthritis
Source: PLoS One. 2014 Jun 2;9(6):e97501. doi: 10.1371/journal.pone.0097501 (PMC4041724; doi:10.1371/journal.pone.0097501)
Supplement: Table S2 — The potential biomarkers of RA found from metabolite analysis of synovial fluid with and without controlling gender ratios of RA and non-RA patients. (DOC) [file pone.0097501.s004.doc]

**Table S2.** The potential biomarkers of RA found from metabolite analysis of synovial fluid with and without controlling gender ratios of RA and non-RA patients.

| Potential biomarkers of RA  without controlling gender ratio^a^ | Potential biomarkers of RA  with controlling gender ratio^b^ |
| --- | --- |
| succinate | succinate |
| octadecanol | acetophenone NIST |
| asparagine | hydroxylamine |
| terephthalate | octadecanol |
| salicylaldehyde | asparagine |
| glutamine | ethanolamine |
| citrulline | lysine |
| tyrosine | citrulline |
| uracil | palmitoleic acid |
| lysine | isopalmitic acid |
| ribitol | salicylaldehyde |
| tryptophan | terephthalate |
| xylose | tyrosine |
| ribose | serine |
| isopalmitic acid | glycerol |
| glycerol | uracil |
| myristic acid |  |
| palmitoleic acid |  |
| hydroxylamine |  |
| ethanolamine |  |

^a^Gender ratio not controlled (13 RA and 25 non-RA patients).

^b^Gender ratio controlled (13 RA and 5 non-RA patients).
